# Diagnosis and incidence risk of clinical canine monocytic ehrlichiosis under field conditions in Southern Europe

**DOI:** 10.1186/s13071-014-0613-4

**Published:** 2015-01-06

**Authors:** Magalie René-Martellet, Isabelle Lebert, Jeanne Chêne, Raphaël Massot, Marta Leon, Ana Leal, Stefania Badavelli, Karine Chalvet-Monfray, Christian Ducrot, David Abrial, Luc Chabanne, Lénaïg Halos

**Affiliations:** Université de Lyon, VetAgro Sup Campus vétérinaire de Lyon, Jeune équipe Hémopathogènes vectorisés, 1 avenue Bourgelat, 69280 Marcy L’Etoile, France; Merial S.A.S, 29 avenue Tony Garnier, 69007 Lyon, France; INRA, UR 346 Epidémiologie Animale, 63122 Saint-Genès-Champanelle, France; VetAgro Sup campus vétérinaire de Lyon, 1 avenue Bourgelat, 69280 Marcy l’Etoile, France

**Keywords:** *Ehrlichia canis*, Clinical canine monocytic ehrlichiosis, Spain, Portugal, Italy, Vector-borne diseases, Dog, Incidence risk

## Abstract

**Background:**

Canine Monocytic Ehrlichiosis (CME), due to the bacterium *Ehrlichia canis* and transmitted by the brown dog tick *Rhipicephalus sanguineus,* is a major tick-borne disease in southern Europe. In this area, infections with other vector-borne pathogens (VBP) are also described and result in similar clinical expression. The aim of the present study was to evaluate the incidence risk of clinical CME in those endemic areas and to assess the potential involvement of other VBP in the occurrence of clinical and/or biological signs evocative of the disease.

**Methods:**

The study was conducted from April to November 2011 in veterinary clinics across Italy, Spain and Portugal. Sick animals were included when fitting at least three clinical and/or biological criteria compatible with ehrlichiosis. Serological tests (SNAP®4Dx, SNAP®Leish tests, Idexx, USA) and diagnostic PCR for *E. canis, Anaplasma platys*, *Anaplasma phagocytophilum, Babesia* spp*, Hepatozoon canis* and *Leishmania infantum* detection were performed to identify the etiological agents*.* Ehrlichiosis was considered when three clinical and/or biological suggestive signs were associated with at least one positive paraclinical test (serology or PCR). The annual incidence risk was calculated and data were geo-referenced for map construction. The probabilities of CME and other vector-borne diseases when facing clinical and/or biological signs suggestive of CME were then evaluated.

**Results:**

A total of 366 dogs from 78 veterinary clinics were enrolled in the survey. Among them, 99 (27%) were confirmed CME cases, which allowed an estimation of the average annual incidence risk of CME amongst the investigated dog population to be 0.08%. Maps showed an increasing gradient of CME incidence risk from northern towards southern areas, in particular in Italy. It also suggested the existence of hot-spots of infections by VBP in Portugal. In addition, the detection of other VBP in the samples was common and the study demonstrated that a dog with clinical signs evocative of CME is as likely to be positive to *Ehrlichia canis* as to another VBP.

**Conclusions:**

The study confirms the endemicity of CME in southern Europe and highlights the difficulties encountered by veterinarians to differentiate CME from other vector-borne diseases under field conditions.

## Background

Canine Tick Borne Diseases are emerging all over Europe and the burden of those transmitted by the brown dog tick *Rhipicephalus sanguineus* is a major concern [1,2]. Among the latest, canine monocytic ehrlichiosis (CME), caused by the bacterium *Ehrlichia canis*, is one of the tick-borne diseases associated with the most marked clinical expression in dogs. CME results in a variety of acute, chronic or subclinical syndromes with different phases of the disease course and multiple clinical manifestations. In the field, diagnosis of CME may be complicated by the possible occurrence of co-infections with other vector-borne pathogens (VBP), including *Anaplasma platys, Babesia canis, B. vogeli, B. microti*-like (previously referred to as *Theileria annae*)*, Hepatozoon canis* and *Leishmania infantum -* some sharing the same vector - that may result in “altered clinical disease manifestations” [3-6]. Therefore, the diagnosis of the disease can be challenging for practicing veterinarians [7]. According to the consensus of the infectious disease group of the American College of Veterinary Internal Medicine (ACVIM) [8], confirmed cases of CME are defined as dogs presented with (i) evocative clinical signs and for which (ii) positive tests are obtained, either by serology and/or by PCR.

As its tick vector *R. sanguineus*, CME has a wide distribution in the world in particular under tropical, subtropical or Mediterranean climates. It is considered enzootic in southern Europe [1]. Although several studies were carried out to evaluate seroprevalence of *E. canis* infection in this area, none have attempted to assess the incidence of the disease in canine populations living there [9].

Thus, the objectives of the study were (i) to evaluate the incidence risk of CME in veterinary clinics and (ii) to calculate the probabilities of CME and other vector-borne diseases in dogs showing evocative signs of CME in endemic areas of southern Europe.

The study was based on the systematic enrollment of sick dogs presented with suspicion of CME in veterinary clinics from Spain, Italy and Portugal and the realization of serological and/or PCR tests for *E. canis* and/or other VBP antibodies or antigens and/or DNA detections. The criteria defined by the expert group of the ACVIM were used for the definition of a case of CME and incidence risk of CME was evaluated for each clinic and was used to construct distribution maps.

## Methods

### Geographical and temporal framework

The study was conducted between April and November 2011, during the seasonal onset of the tick vector *R. sanguineus* under Mediterranean climates. Since the incubation of CME following tick transmission is short (from 2 to 4 weeks) [6], this time period was considered to correspond also to the window of occurrence of the disease. Veterinary clinics were selected on a voluntary basis in Portugal, Spain and Italy taking attention to cover homogeneously each country.

The survey was anonymized, veterinary practitioners participated on a voluntary basis and no specific procedure was undertaken on animals. No approval by an ethics committee was required for the applied methodology.

### Dogs inclusion

*Dogs selection by veterinarians*: veterinarians were asked to select sick dogs presented to their practice when fitting at least three criteria compatible with CME among a list of clinical and/or biological signs suggestive of the disease (Table 1), i.e. dogs with a documented suspicion of CME. The association of at least three signs was required because of the non-specific expression of the disease. For each dog selected, veterinarians had to sample blood for diagnostic confirmation and to fill a registration form gathering anamnesis, description of clinical signs, results of additional tests if any and additional epidemiological information such as the travel history, the life style (indoor, outdoor) and the frequency of contacts with other animals.

**Table 1:** **Clinical and biological signs suggesting canine monocytic ehrlichiosis used for dogs selection**

**Clinical signs**	**CBC** ^**1**^ **abnormalities**	**Biochemistry abnormalities**
Fever	Moderate to severe thrombocytopenia	Hypoalbuminemia
Depression, lethargy, weakness	Anaemia	Hyperglobulinemia
Anorexia	Leukopenia	Increase in
Lymphadenomegaly	Lymphocytosis	- alanine aminotransferase (ALT)
Splenomegaly		- alkaline phosphatase (ALP)
Haemorrhagic tendencies *(including dermal petechiae and ecchymoses, epistaxis)*		- C-reactive protein (CRP)
		- alpha 1-acid glycoprotein (AAG)
Pale mucous membranes		
Weight loss		
Ophthalmological lesions *(including anterior uveitis, chorioretinitis, papilledema, retinal haemorrhage, retinal perivascular infiltrates, bullous retinal detachment)*		
Neurological disorders		

^1^Complete blood count.

*Criteria used for dogs inclusion in the survey*: Dogs selected by veterinarians were finally included in the survey when associating:

- Suspicion of CME based on three clinical and/or biological suggestive signs
- At least an *E. canis* serological test performed at the clinic or a blood sample available for PCR analyses.

No specific agreement was required as the management of the dogs followed the classical process of a field veterinary consultation.

### Sampling methods and serological tests performed at the clinic

Venous blood samples (whole blood and blood on EDTA) were obtained from the cephalic or jugular veins of dogs and stored at 5°C +/− 1°C until analysis. SNAP®4Dx test (Idexx, Westbrook, USA) was used on whole blood or sera to detect antibodies of *E. canis* but also antibodies of *Borrelia burgdorferi*, *Anaplasma* spp and antigens of *Dirofilaria immitis*. Moreover, SNAP®Leish test (Idexx, Westbrook, USA) was performed on samples to allow detection of antibodies of *Leishmania infantum*.

### PCR analyses

Blood samples from each dog were then sent to the Laboratory of Parasitology and Parasitic Diseases of VetAgro Sup (Marcy l’Etoile, France) for PCR analyses. DNA was extracted from blood samples as previously described [10] and the quality of each extracted DNA was assessed by PCR amplification of mitosin gene specific for dogs to confirm the presence of dog DNA and the absence of PCR inhibition [11]. Then, multiplex PCR amplifications were performed from each DNA blood sample to detect DNA of *E. canis*, *A. platys*, *A. phagocytophilum, Babesia* spp, *H. canis* and *Leishmania* spp using primers previously designed (Table 2). The amplification reactions were carried out in a thermocycler (Biometra T gradient, Goettingen, Germany) in a total of 25 μL containing 1.25 μL of 2 μM of each primer, 12.5 μL of 2× Type-it Multiplex PCR Master mix (Qiagen Multiplex PCR Kit, Qiagen, Hilden, Germany), 1 μL DNA and 9 μL of RNase free water and the following conditions were applied: 95°C 5 min, 35 cycles at 95°C 30 s, 61°C 90 s, 72°C 30 s and 60°C 30 min. A negative control (reaction mix without DNA) and a positive control (mix of pathogens DNA and dog DNA) were systematically included in parallel. For all *Babesia* spp positive samples, species were characterized using a RFLP-PCR method using TaqI and HinfI enzymes as previously described [12,13].

**Table 2:** **Primers used for vector-borne pathogens detection by PCR in the study**

**Gene target**	**PCR target**	**Name**	**Primer sequence**	**Fragment length**	**Reference**
Mitosin gene	*Canis familiaris*	CAN-F	5′-CTTGTCACGGTAAGGTTC-3′	290-bp	[14]
		CAN-R	5′-CTGATGTATTTCCTGCACCAAG-3′		
Vir-B9 protein gene	*Ehrlichia canis*	Ehr1401F	5′-CCATAAGCATAGCTGATAACCCTGTTACAA-3′	380-bp	[15]
		Ehr1780R	5′-TGGATAATAAAACCGTACTATGTATGCTAG-3′		
GroEL gene	*Anaplasma platys*	GroAplatys-35 s	5′-AGCGTAGTCCGATTCTCCAGTTTT-3′	515-bp	[16]
		GroAplatys-550as	5′-TCGCCGTTAGCAGAGATGGTAG-3′		
AnkA gene	*Anaplasma phagocytophilum*	AnkAP-2074s	5′-GGCAAATGAGGCAAGTAACC-3′	741-bp	[16]
		AnkAP-2815as	5′-GCCACTACCCAAGGATGATAG-3′		
18S rDNA	*Babesia* spp	Ba103F	5′-CCAATCCTGACACAGGGAGGTAGTGACA-3′	612-bp	[15]
		Ba721R	5′-CCCCAGAACCCAAAGACTTTGATTTCTCTCAAG-3′		
18S rDNA	*Hepatozoon canis*	Hep F	5′-ATACATGAGCAAAATCTCAAC-3′	666-bp	[17]
		Hep R	5′-CTTATTATTCCATGCTGCAG-3′		
18S rDNA	*Leishmania* spp	R221	5′-GGTTCCTTTCCTGATTTACG-3′	603-bp	[18]
		R332	5′-GGCCGGTAAAGGCCGAATAG-3′		

### Definition of CME cases

As the presence of evocative clinical signs was one of the criteria of selection, all dogs included in the study had *de facto* clinical findings compatible with ehrlichiosis.

In agreement with the consensus opinion of the infectious disease group of the ACVIM on CME diagnosis [8], a CME confirmed case was defined in the study as a dog with clinical signs suggestive of the disease (clinical suspicion) and for which at least one of the two biological tests (*E. canis* serology and/or PCR) was positive.

### Dog population

In order to calculate the incidence risk of the disease, *i.e*. the percentage of the dog population that contracted CME during the period of the study, reference data about the dog populations at risk were required. For that purpose, the number of dogs referring to each clinic involved in the study was collected. In Italy and Portugal, this number was communicated by the veterinarian surgeons. In Spain, this number was not available. Therefore, it was estimated as the number of companion animal veterinarians in the clinic multiplied by the average number of dogs per companion animal veterinarian in the country as previously described [19].

### Computation of the annual incidence risk

The number of CME cases occurring during the study period in the clinic was considered to be equivalent to the annual number of CME cases since the study took place during the whole period of vector activity. As previously explained, CME was considered when at least one of the two biological tests (*E. canis* serology and/or PCR) was positive. For dogs negative for one test and undetermined for the other, a probability of CME was estimated for incidence risk calculation using the dataset from the dog population for which both tests were available:

- For dogs with PCR negative and serology undetermined, the probability of CME was given by the proportion of dogs with positive serology among dogs negative to *E. canis* by PCR.
- For dogs with serology negative and PCR undetermined, the probability of CME was given by the proportion of dogs with positive PCR among dogs negative to *E. canis* by serology.

The annual CME incidence risk (CME IncR_clin_) in each clinic was calculated as the ratio of the total number of dogs positive for CME in the clinic to the dog population referring to this clinic following the formula:

$$ CME\ Inc{R}_{clin}=\frac{\mathrm{n}\_\mathrm{CMEpos}}{\mathrm{n}\_\mathrm{DOG}1} $$

where n_CMEpos is the total number of dogs positive for CME in the clinic, including the dogs with unknown status for which a probability of CME was attributed as explained above; n_DOG1 is the dog population in each clinic.

95% confidence intervals of the annual CME incidence risk were calculated in each country using Exact Binomial test in R software [20]. As the number of cases was low in each clinic, a Fisher’s Exact test was performed to compare the incidence risk of CME of each clinic that participated to the study to the average incidence risk observed in the three countries. A probability p value < 0.05 was considered as statistically significant.

### Map building

The geo-referencing of 57 administrative regions of Spain, Italy and Portugal was performed with ESRI Data & Maps (2005, Redlands, USA). The location of the clinics with geographical coordinates of the locality was obtained using Google Earth. Mapping used GIS software (QGIS v2.0.1 - Dufour) to observe distribution patterns of the clinics and to perform statistical analysis of the incidence risk of CME. A smoothed interpolated map of the CME incidence risk was produced using an Inverse Distance Weighting (IDW) interpolation method. IDW in QGIS was set at 2.0. The colored scale in the raster obtained was proportional to the value of the incidence rates.

### Probability of diagnosis of CME and other VBD

For a few dogs, information regarding the results of the tests against some of the targeted VBP was missing. In order to estimate probability of diagnosis of CME and other VBD, we used the subset of dogs with complete data for all tests.

The probabilities of diagnosis of CME (pCME) or another VBD (pVBD) in the absence of CME, when facing clinical or biological signs consistent with CME were estimated as follows:

$$ {p}_{CME}=\frac{\mathrm{n}\_\mathrm{CMEpos}}{\mathrm{n}\_\mathrm{DOG}2} $$

$$ {p}_{VBD}=\frac{\mathrm{n}\_\mathrm{VBDpos}}{\mathrm{n}\_\mathrm{DOG}2} $$

where *p*_*CME*_ and *p*_*VBD*_ are respectively the probability of diagnosis of CME and the probability of diagnosis of another vector-borne disease when negative for CME; n_CMEpos and n_VBDpos are the number of dogs positive for CME and the number of dogs positive for other vector-borne diseases and negative for ehrlichiosis, respectively; n_DOG2 is the number of dogs for which all the diagnostic methods where applied.

## Results

### Questionnaire records and blood collections

**Figure 1:**
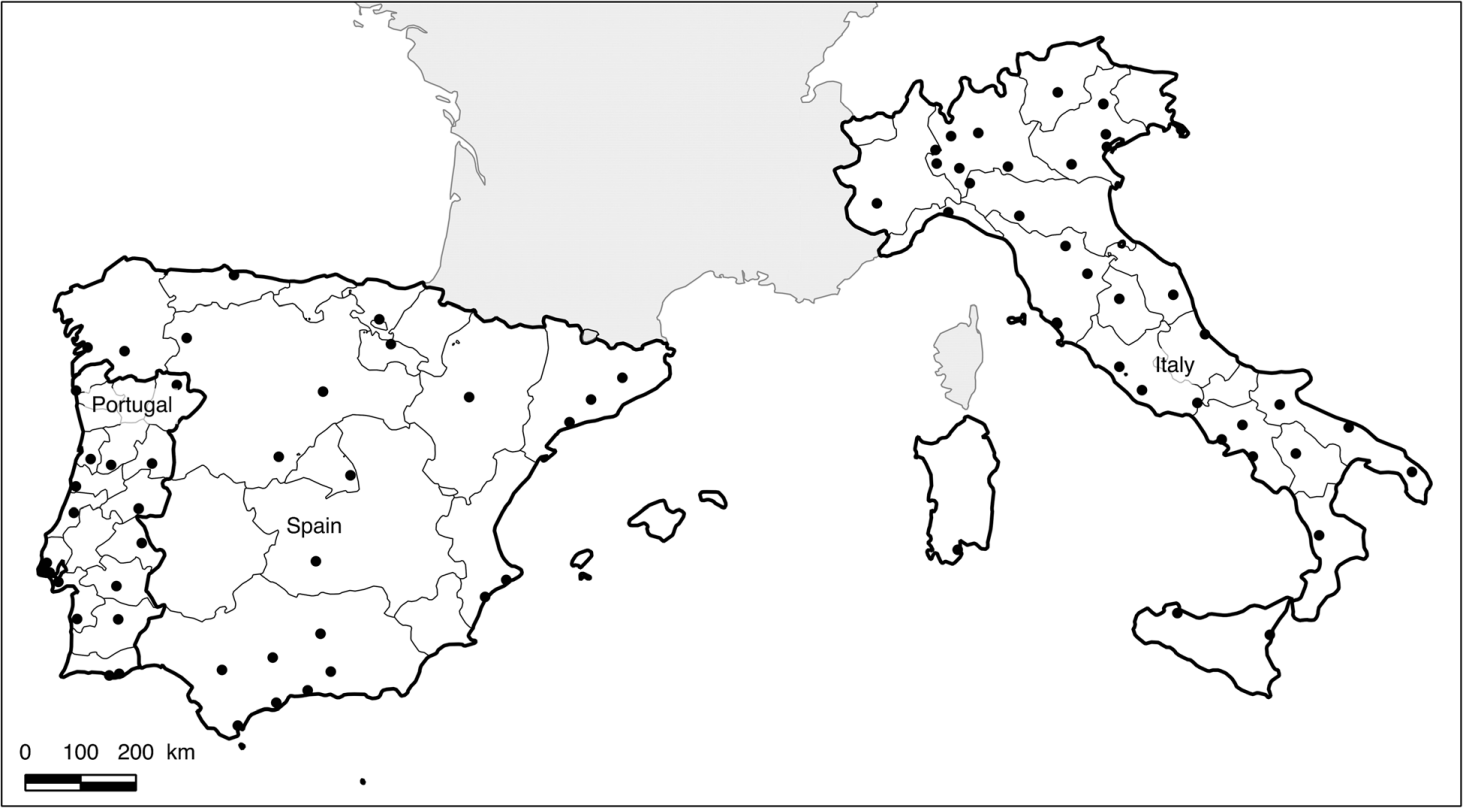
**Map of distribution of the veterinary clinics that took part to the study.** The map shows provinces in Portugal, Spain and Italy and locations where veterinary clinics, represented by dots, were integrated in the study and from which cases of canine monocytic ehrlichiosis were obtained. The map confirms the fairly homogenous distribution of the sampling sites.

From April to June 2011, 78 veterinary clinics (23 in Spain, 37 in Italy and 18 in Portugal) took part to the study with a fairly homogeneous distribution throughout the countries. In Spain sampling sites were scarcer (Figure 1). Within those 78 clinics, 366 dogs were included in the survey. Among them, 102 were from Spain, 144 from Italy and 120 from Portugal.

### Detection of Ehrlichia and other Vector-Borne Pathogens (VBP)

From these 366 dogs included, 356 were tested using SNAP®4Dx test, 340 using SNAP®Leish test, 317 were screened by PCR. Among the 366 dogs, 291 were tested using all diagnostic methods. Results of VBP detection by PCR and serology are summarized in Table 3.

**Table 3:** **Results of vector-borne pathogens (VBP) detection**

**Targeted VBP**	**Specific PCR**	**Serology**
	**Number of positive samples/number of tested samples**	**Number of positive samples/number of tested samples**
*Ehrlichia canis*	35/317 (11%)^1^	92/356 (26%)
*Leishmania infantum*	20/317 (6%)^2^	66/340 (19%)
*Anaplasma* spp.	ND	31/356 (9%)
*A. platys*	24/317 (8%)^3^	ND
*A. phagocytophilum*	0/317 (0%)	ND
*Hepatozoon canis*	24/317 (8%)	ND
*Babesia* spp.	15/317 (5%)	ND
*Dirofilaria immitis*	ND	8/356 (2%)^4^
*Borrelia burgdorferi*	ND	4/356 (1%)

^1^Including 28 dogs also positive by serology.

^2^Including 12 dogs also positive by serology.

^3^Including 5 dogs also positive for *Anaplasma* sp. by serology.

^4^Antigens detection.

ND: Not done.

Serology allowed detection of antibodies of *E. canis* in 26% of the serum tested and specific DNA of *E. canis* was detected in 11% of the blood samples tested. Thus, a total of 99 dogs were positive either by PCR or serology to *E. canis*.

*L. infantum* was detected in 19% of the serum tested and its specific DNA was identified in 6% of the samples giving a total of 74 dogs positive either by PCR or by serology to *L. infantum*. Antibodies against *Anaplasma* sp. were detected in 9% of the sera. Only DNA of *A. platys* was identified in 8% of the blood samples whereas no dogs were found infected with *A. phagocytophilum* using PCR analysis. Thus, a total of 50 dogs were found positive to *Anaplasma* sp. either by PCR or by serology. Four dogs had a serological test positive for *B. burgdorferi* (1%) and antigens of *D. immitis* were detected in 8 dogs (2%).

Multiplex PCR allowed detection of DNA of *H. canis* in 8% dogs.

*Babesia* spp were detected by PCR in 5% dogs tested. Species characterization using RFLP identified *B. canis* in 5 dogs (4 from Spain and 1 from Portugal), *B. vogeli* in 6 dogs (2 from Portugal, 2 from Spain and 2 from Italy) and *B. microti-*like in 4 dogs from Spain (2 in Galicia, the endemic region for *B. microti-*like and 2 in Southern Spain).

**Figure 2:**
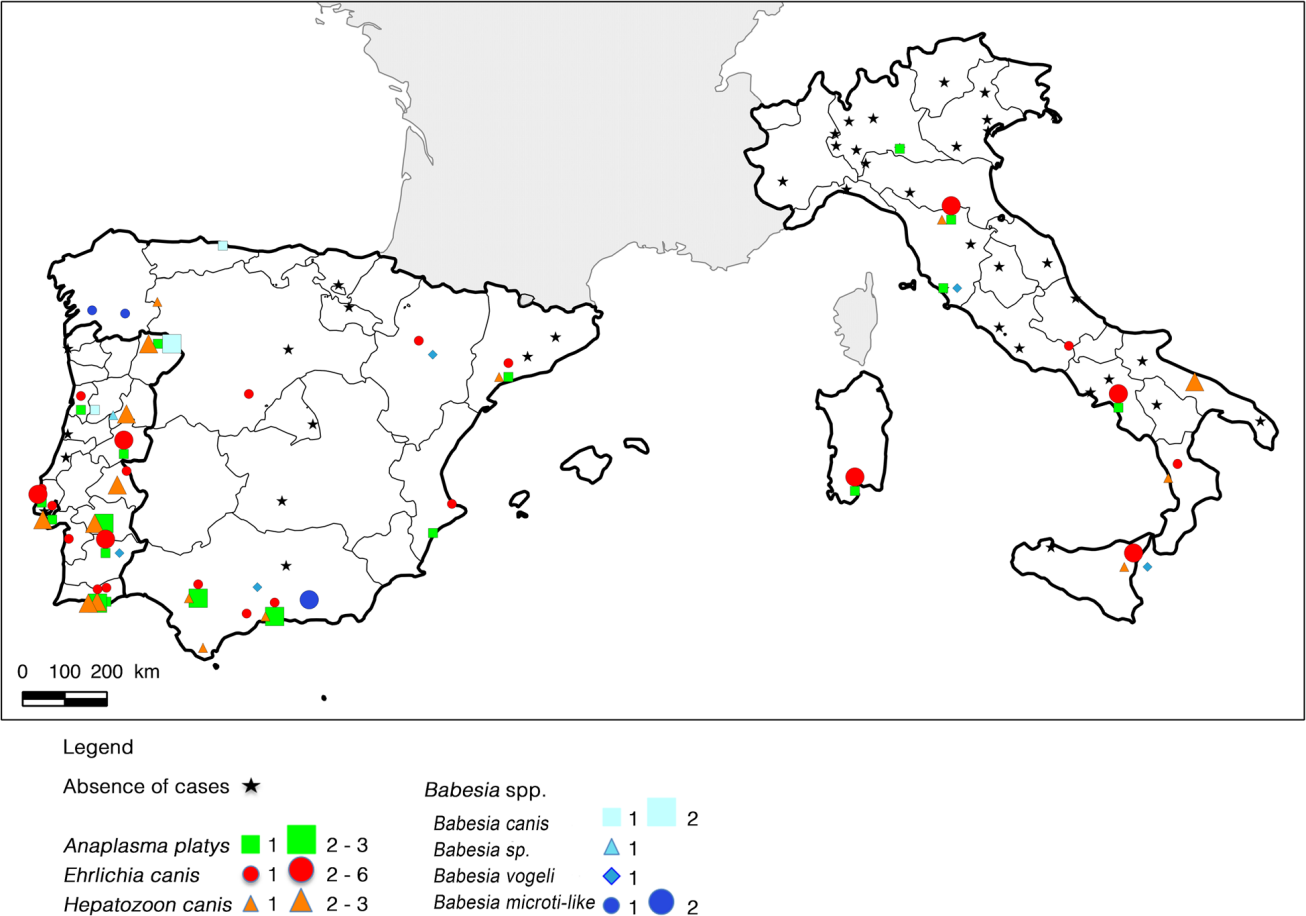
**Geographical distribution of Tick-Borne Pathogens (TBP) detected in dog blood samples by PCR.** The figure shows provinces in Portugal, Spain and Italy and locations where dogs with clinical suspicion of CME were detected positive to one or several of the TBP screened by PCR. Stars correspond to locations where no dog was found positive to TBP by PCR. Portugal was the country where the greatest number of TBP was evidenced.

A map showing the geographical origin of all dogs positive to tick-borne pathogens detected in the study is presented in Figure 2.

In addition, the high number of samples positive for several VBP is noticeable. Among the 99 dogs positive for *E. canis*, serological or PCR detection of another VBP was done in almost the half of them (42/99). The most frequently VBP found associated with *E. canis* were *Anaplasma* spp. (N = 14; 4%) and *L. infantum* (N = 14; 4%) detected by serology, followed by *H. canis* (N = 10; 3%), *A. platys* (N = 8; 2%), *Babesia* spp (N = 6; 2%) and *L. infantum* (N = 6; 2%) detected by PCR whereas co-detection of *B. burgdorferi* and *D. immitis* were evidenced in 2 (0.5%) and 1 (0.3%) cases respectively.

Out of the 366 dogs included, 224 were negative to *E. canis.* Among them, 78 (21% of the 366 dogs enrolled) were positive to other VBP (except *E. canis*) by serology and/or PCR. In 62 dogs, blood samples were positive to a single VBP (*Leishmania* spp in 32 dogs, *Anaplasma* spp in 17 dogs, *Babesia* spp in 6 dogs, *H. canis* in 5 dogs, *D. immitis* in 2 dogs). In 10 dogs, several VBP (except *Ehrlichia*) were detected and 6 dogs were positive either to one or several VBP because they were not tested for all VBP.

Finally, 137 out of the 366 dogs included (37%) were negative for all VBP. Final diagnosis, when available revealed other non vector-borne infectious process, tumoral process, autoimmune anaemia, intoxications or non-identified causes.

### CME incidence risk and geographical distribution

Among the 366 dogs included in the study, 99 were considered as CME cases and 224 were not. Forty-three dogs had an unknown status for CME because they were not tested by both methods: for 7 dogs, serology was not determined and PCR was negative, and for 36 others, PCR was not determined and serology was negative. In order to avoid a bias in the incidence risk estimate, the probability of infection of these 43 dogs was estimated as explained in the Methods section:

- For the 7 dogs with PCR negative and serology undetermined, the probability of CME was evaluated to 18.5%, corresponding to the number of dogs positive by serology (N = 51) among the dogs negative by PCR (N = 275). These 7 dogs represented 1.26 supplementary CME cases.
- For the 36 dogs with serology negative and PCR undetermined, the probability of CME was evaluated to 1.8%, corresponding to the number of dogs positive by PCR (N = 4) among the dogs negative by serology (N = 228). These 36 dogs represented 0.72 supplementary CME cases.

The average annual incidence risk of CME in the 78 clinics of the three countries was equal to 0.08% (CI 95%: 0.06 – 0.09%). The average annual incidence risk in Spain was 0.03% (CI 95%: 0.01 – 0.04%), in Italy 0.10% (CI 95%: 0.08 – 0.13%) and in Portugal 0.14% (CI 95%: 0.09 – 0.19%). The annual incidence risk observed in Spain was significantly lower than those in the two other countries (P < 0.05).

**Figure 3:**
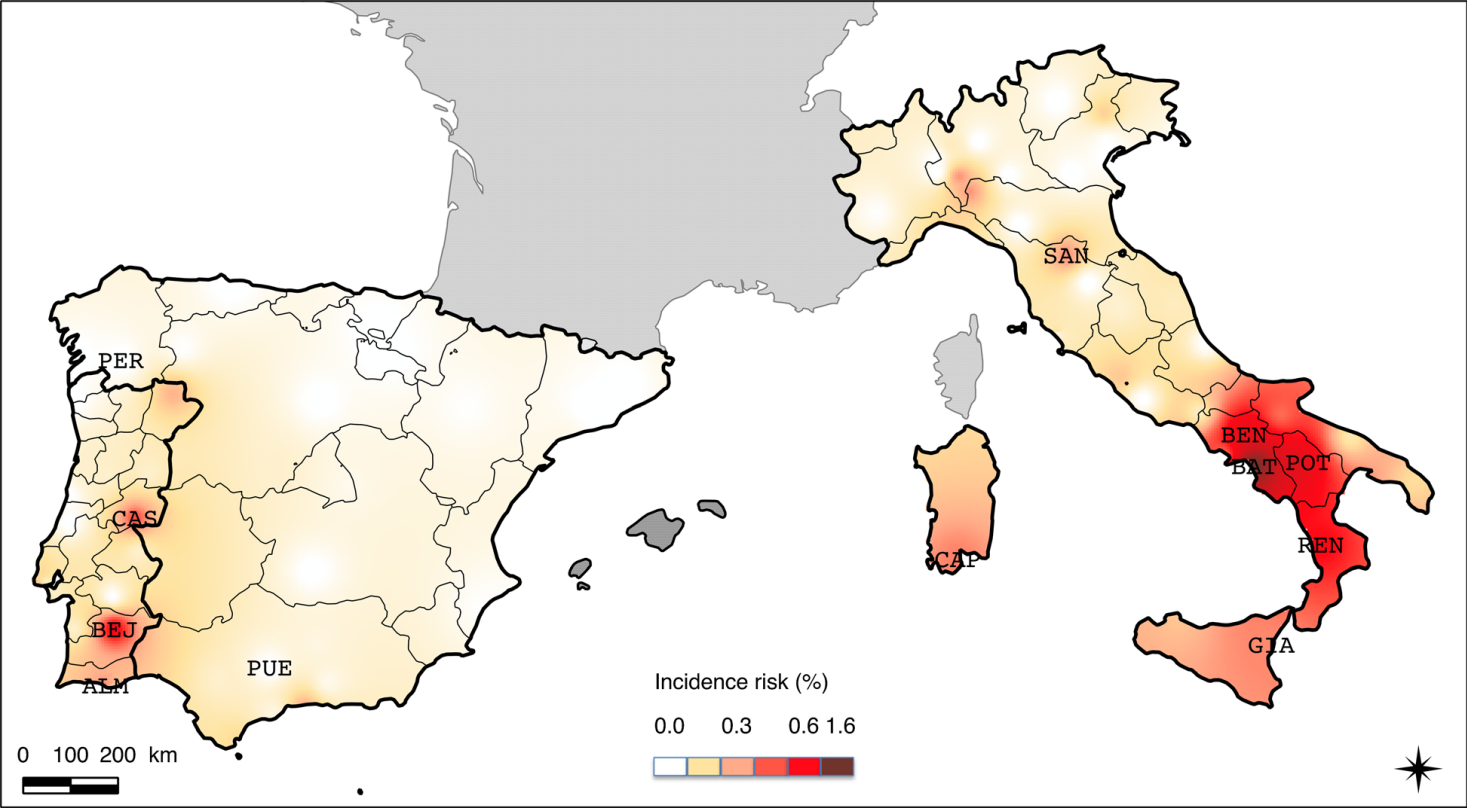
**Interpolated map of annual CME incidence risk in Italy, Spain and Portugal in 2011.** Only towns where clinics had incidence significantly different from the average incidence of the three countries were indicated: PER, Pereiro De Aguiar and PUE, Puente Genil in Spain; SAN, San Piero a Sieve; BAT, Battipaglia; BEN, Benevento; POT, Potenza; CAP, Capoterra; GIA, Giarre; REN, Rende in Italy and CAS, Castello Branco; BEJ, Beja; ALM, Almancil in Portugal.

An interpolated map of annual CME incidence risk in Italy, Spain and Portugal was then produced (Figure 3). An increasing gradient of infection from North to South emerges from this analysis. Incidence risk in southern Italy appears to be higher than in other areas including southern Spain and Portugal. In Spain, the distribution shows also an increasing gradient from central Spain to western and southern borders. In Portugal, a West to East (Spain border) increasing gradient was observed with hot-spots of detections in the southern part of the country.

The annual incidence risks of CME in two clinics in Spain (clinics in the towns Pereiro De Aguiar and Puente Genil, incidence equal to zero) were significantly lower than the average incidence risk in the 78 clinics of the three countries. In Portugal, three clinics had a significantly higher annual incidence risk of CME compared to the average incidence risk of the three countries (in towns Beja, Castello Branco and Almancil with incidence risk of 0.64%, 0.46% and 0.36% respectively). Seven Italian clinics had significantly higher incidence risk than the average incidence risk of the three countries in the towns Battipaglia (1.60%), Potenza (0.80%), Benevento (0.78%), Rende (0.60%), Capoterra (0.37%), Giarre (0.36%), San Piero a Sieve (0.34%).

### Probability of diagnosis of CME by veterinarians

The probability of diagnosis of CME and other VBP infections in dogs with clinical suspicion of CME was computed on the 291 dogs for which results of all diagnosis tests were available (PCR and/or serology for all VBP).

Eighty-two dogs were positive for *E. canis* using at least one of the two diagnostic methods (PCR and/or serology) and, therefore, were considered as confirmed cases of the disease. The probability of diagnosis of CME by veterinarians (*p*_*CME*_*)* when facing a suspicion of CME based on three clinical or biological signs consistent with an infection with *E. canis* was evaluated at 0.28 [0.23; 0.33].

Among dogs negative to *E. canis*, 72 were positive for at least one other VBP, so the probability of diagnosis of other VBD in the absence of CME (*p*_*VBD*_) was evaluated to 0.25 [0.20; 0.30].

A total of 137 dogs were negative to all tests. The probability of a negative diagnosis of CME or other VBD was 0.47 [0.41; 0.53].

## Discussion

Numerous studies on the seroprevalence of VBD of pets in endemic areas have been performed during the last decades [9,21-23]. However, studies evaluating incidence risk of CME are rare, in particular studies presenting rates in reference to a dog population [9]. The study conducted in 2011 allowed to collect samples from 366 cases of CME suspicion in 78 veterinary clinics from Spain, Portugal and Italy. According to the results of serological and molecular tests, an overall annual incidence risk of the disease in those countries was calculated at 0.08% in the three countries with important geographic variations (from 0 to 1.6%). In addition, the identification of other vector-borne pathogens in the samples was extremely frequent and the study demonstrated that a dog with clinical signs evocative of CME is as likely to be positive to *Ehrlichia canis* as to another VBP including *Anaplasma platys, Babesia canis, B. vogeli, B. microti*-like*, Hepatozoon canis* or *Leishmania infantum.*

To support the clinical suspicion, veterinarians were asked to perform serological tests at the clinic and to send blood for PCR confirmation in laboratory. Among the 366 included cases, 291 (79.5%) were analyzed using all diagnostic methods. This relatively high percentage of blood analyzed confirms the strong involvement of veterinarians that accepted to participate in the study. However, we could not exclude that some veterinarians did not fulfill perfectly the protocol and did not submit all clinical suspects; in this case, it might have induced an underestimate of the incidence risk of CME.

As previously noted, CME is a complex disease with several clinical phases characterized by different expressions, which means diagnosis may be extremely challenging [7]. In the present study, we chose to include only dogs with clinical suspicions of CME, i.e. with expressed clinical signs, and to consider a positive diagnosis of CME when at least one biological test was positive for *E. canis* either by serology and/or by PCR*.* This postulate was based on recommendations from experts [8] but may have generated biases.

PCR generally allows detection of dogs in the clinical (=”acute”) phase of the disease. On the contrary, serology allows detections of dogs in later phases such as sub-clinical, chronic or recovering. In the study, the selection of dogs positive either by serology and/or by PCR and the selection of dogs with clinical and/or biological signs suggest the possibility of selection of dogs in any stages of the disease. However, using this method of recruitment, it is possible that some dogs, in a sub-clinical phase (very slight clinical signs) were not selected by veterinarians and thus were excluded from the study at an early stage. A qualitative test (SNAP®4Dx test, Idexx, USA) was employed because of its ease of use in the field and its capacity to detect several VBP with a single test. This test was calibrated by the manufacturer to be positive at a titer of approximately 1:100 or greater [8]. Therefore, the capacity of antibodies detection by this test is higher than the threshold value (1:80) below which cases must be considered as doubtful according to the consensus. Moreover, according to the same experts group, “titers do not correlate with the duration of infection or the severity of disease” and consequently do not allow to conclude to the “ill”, “just exposed” or “infected” status of the dogs. In addition, dogs negative by serology were, in most cases in the study, tested by PCR that is one of the 3 methods recommended by the consensus for CME doubtful cases confirmation. Thus, it was decided to consider the qualitative SNAP®4Dx test sufficient for CME diagnosis in the context of the study. It is possible however that some dogs under seroconversion process (i.e. with an IFA titer under 1:100 at the time of the test but with a possible higher titer few days later) were not detected by the method. Finally, the study was conducted from April to November with the exclusion of winter months when the expression of acute disease is limited. It excluded therefore few dogs with delayed signs of chronic CME. All these considerations could have led to an underestimation of the risk in particular in places where incidence is high.

On the other hand, we cannot exclude that some of the dogs considered as positive because of a positive serology, had actually a previous contact with *E. canis* (recovery or sub-clinical phase) and had clinical signs caused by another disease, including another VBP infection. In this case, it could have led to an overestimation of CME risk. Nevertheless, despite those elements of uncertainty, the present study offers an interesting picture of the field expression of CME in endemic areas.

The geographical coverage was homogeneous in Portugal and Italy but rather sparse in Spain. This may limit the accuracy of the results in the country. Geographic heterogeneities due to the presence of mountains, rivers or sea boarders were not taken into account in the interpolation method. However, the sampling method allowed a raw coverage of the geographical diversity of the three countries and the resulting maps illustrated contrasted epidemiological situations regarding CME and other VBD.

The study suggests the existence of gradients and hot-spots of CME infections. In particular, incidence risk in southern Italy appears to be higher than in other areas including southern Spain and Portugal. A clear increasing gradient of CME incidence from North to South emerges from this analysis.

In Spain, mean incidence risk was relatively low (0.03% ranging from 0 to 0.27% between clinics) with only sporadic cases or small foci. Cases were mainly detected in the southern part of the country, essentially near the coasts, whereas in a previous survey, seropositive dogs were mostly detected near the northern coasts [22]. These differences in distribution may be due to either the low number of veterinary clinics that took part in the study in Spain or the method (dog selection or diagnosis) used. However, Spain is divided in two phytogeographic regions, the Eurosiberian region in the northern part, characterized by typically oceanic climate and vegetation, and the Mediterranean region, in the rest of the country, with hot summers and cold winters in the mountains of central Spain and hot but milder climate around the coasts [24]. Knowledge on ecological and climatic preferences of the vector tick species, *R. sanguineus* (warm climate with mild winter) [25], and previous reports [26], suggest it may be more abundant in the hot milder areas of southern coasts than in other areas of the country and supports our results.

In Portugal, mean incidence risk was higher (0.14%, ranging from 0 to 0.64% between clinics); three towns situated in central and southern Portugal along the Spain border (Beja, Castello Branco and Almancil towns) had higher incidence risks than elsewhere in the country and appeared hot-spots of CME infection. A previous survey showed that Portugal is a highly endemic country for VBD [21]. Regarding CME distribution, results of our study correspond to those previously published with higher positivity rates in southern areas compared to northern [21]. *R. sanguineus* is described as the most prevalent tick species throughout all the regions of the mainland [21].

In Italy, *E. canis* was detected throughout the country as well as in Sardinia and Sicily (mean incidence risk 0.10%, ranging from 0 to 1.6% between clinics). A clear increasing gradient of detection was evidenced from the North to the South. This distribution is similar to those previously published and corresponds to the known distribution of the vector *R. sanguineus* in the country [23].

In addition to the detection of *E. canis*, serological or PCR tests were performed targeting other VBP known to circulate in southern Europe, susceptible to infect dogs with similar clinical expression and/or sharing the same vector. It included serological detection of other pathogens targeted by the SNAP®4Dx test (*Anaplasma* spp., *B. burgdorferi*, and *D. immitis* (detection of antigens)) and *L. infantum.* In order to allow an accurate differential diagnosis, the detection of specific DNA from *A. platys*, *A. phagocytophilum, Babesia* spp, *H. canis* and *Leishmania* spp. was also conducted on the blood samples. These tests confirmed the high level of circulation of those VBP in those endemic areas of southern Europe resulting in a high rate of detection of other VBP among the *E. canis* positive dogs but also in a high rate of other VBP detection among the dogs selected for clinical suspicion of CME and finally negative for *E. canis*. Interestingly, *A. platys*, which is usually considered as less pathogenic than *E. canis* with essentially subclinical expression was identified by PCR in mono-infection of at least 9 dogs included in the study.

According to the study, when facing at least three clinical or biological signs consistent with CME, the probability to find PCR or serology evidence of CME is equal to 0.28. In the absence of CME, the probability to find evidence of another VBD is 0.25. These findings highlight the difficulties encountered by veterinarians to conduct a differential diagnosis between the different vector-borne diseases in endemic areas based on clinical suspicion under field conditions. If a vector-borne pathogen was identified in half of the cases, the cause of the disease was not linked to vector-borne infectious process in the other half illustrating that diagnosis of those polymorphic diseases in the field remains challenging. Future studies should focus on identifying reliable clinical and/or biological parameters to help veterinarians in their diagnosis procedure.

A distribution map was constructed in order to localize the place where DNA from other tick-borne pathogens were collected. Interestingly, the detection of several pathogens appeared to be more concentrated in hot-spots that correspond to those identified for CME especially along the eastern border of Portugal.

## Conclusions

This survey conducted in 2011 allowed evaluation of incidence risk of CME in 3 endemic countries, Italy, Spain and Portugal, and suggested the probable existence of hot-spots of infections and gradients of distribution. Similar studies should be conducted in the future in other countries of Europe in order to enhance knowledge on epidemiology of the disease but also to assess the putative progression of CME and its vector *R. sanguineus* to the North. The study highlights also the high frequency of infections and co-infections by several VBP in those endemic areas and demonstrates the complexity of the diagnosis of those diseases in practice.
